# Pathognomonic features of *Pasteurella multocida* isolates among various avian species in Sharkia Governorate, Egypt

**DOI:** 10.1007/s11274-023-03774-2

**Published:** 2023-10-09

**Authors:** Azza S. El-Demerdash, Rehab E. Mowafy, Hanan A. Fahmy, Ahmed A. Matter, Mohamed Samir

**Affiliations:** 1 Laboratory of Biotechnology, Department of Microbiology, Agriculture Research Centre (ARC), Animal Health Research Institute (AHRI), Zagazig, 44516 Egypt; 2 Department of Pathology, Agriculture Research Centre (ARC), Animal Health Research Institute (AHRI), Zagazig, 44516 Egypt; 3 Agriculture Research Centre (ARC), Animal Health Research Institute (AHRI), Dokki, Giza, 12618 Egypt; 4 Agricultural Research Centre, Animal Health Research Institute, Reference Laboratory for Veterinary Quality Control On Poultry Production, Gamasa, 12618 Egypt; 5 Department of Zoonoses, Faculty of Veterinary Medicine, Zagazig, 44511 Egypt

**Keywords:** Avian species, Epidemiological mapping, Histopathological features, Molecular characterization, Multidrug resistance, *Pasteurella multocida*

## Abstract

The present study aimed to isolate *Pasteurella multocida (P. multocida*) from pulmonary cases in several avian species and then investigate the histopathological features, antimicrobial resistance determinants, virulence characteristics, and risk factors analysis of the isolates in each species in correlation with epidemiological mapping of pasteurellosis in Sharkia Governorate, Egypt. The obtained data revealed a total occurrence of 9.4% (30/317) of *P. multocida* among the examined birds (chickens, ducks, quails, and turkeys). The incidence rate was influenced by avian species, climate, breed, age, clinical signs, and sample type. Antimicrobial susceptibility testing revealed that all isolates were sensitive to florfenicol and enrofloxacin, while 86.6 and 73.3% of the isolates displayed resistance to amoxicillin-clavulanic acid and erythromycin, respectively. All of the *P. multocida* isolates showed a multiple-drug resistant pattern with an average index of 0.43. Molecular characterization revealed that the *oma87, sodA,* and *ptfA* virulence genes were detected in the all examined *P. multocida* isolates. The *ermX* (erythromycin), *blaROB-1* (β-lactam), and *mcr-1*(colistin) resistance genes were present in 60, 46.6, and 40% of the isolates, respectively. Ducks and quails were the most virulent and harbored species of antimicrobial-resistant genes. These results were in parallel with postmortem and histopathological examinations which detected more severe interstitial pneumonia lesions in the trachea and lung, congestion, and cellular infiltration especially in ducks. Epidemiological mapping revealed that the Fakous district was the most susceptible to pasteurellosis infection. Thus, farmers are recommended to monitor their flocks for signs of respiratory disease, seek veterinary care promptly if any birds are sick, and avoid the random usage of antibiotics. In conclusion, this study presents a comprehensive picture of the risk factors in correlation to the pathognomonic characteristics of *P. multocida* infection in poultry sectors to help in developing more effective strategies for prevention and control.

**Supplementary Information:**

The online version contains supplementary material available at 10.1007/s11274-023-03774-2.

## Introduction

Infections with *P. multocida* frequently result in epidemics of fowl cholera, causing enormous financial losses for the global poultry sector (Sid et al. 2015).

The mechanism of disease occurrence in *P. multocida* infections is as follows: the bacterium is inhaled or ingested by the host, attaches to the host’s cells, produces toxins that damage the host’s cells and tissues, and evades the host’s immune system, thus causing disease (Wilson et al. 2002).

The infections caused by *P. multocida* include: pneumonia, septicemia, meningitis and eye infections such as conjunctivitis and keratitis (Christenson et al. 2015; Corchia et al. 2015; Lopez and Martinson 2017; de Cecco et al. 2021).

Clinical symptoms of *P. multocida* isolates range from asymptomatic or moderate chronic upper respiratory distress to acute, pneumonic, and/or disseminated disease (Wilson and Ho 2013). The pathogenesis of *P. multocida* is influenced by a variety of virulence factors, such as genes involved in capsule formation, lipopolysaccharide (LPS), fimbriae and adhesins, toxins, iron-controlled and iron acquisition proteins, sialic acid metabolism, hyaluronidase, and outer membrane proteins (OMPs) (Harper et al. 2006). *P. multocida*'s two important surface elements, capsule, and LPS, serve as its primary foundation for classification. Serologically, the bacterium is classified into five capsular serogroups (A, B, D, E, F) and/or 16 somatic serotypes according to its capsule and/or LPS antigens, respectively (Heddleston and Rebers 1975; Carter 1984). However, these traditional serological typing methods are too complicated to be used, as the preparation of the high-sensitive antiserum required for these methods is very difficult (Peng et al. 2016). Therefore, molecular typing methods have been developed to help assign *P. multocida* into five capsular genotypes (A, B, D, E, F) (Townsend et al. 2001) and eight LPS genotypes (L1–L8) (Harper et al. 2015).

There is strong evidence, based on a wide range of molecular research, that avian isolates of *P. multocida* are incredibly varied (Davies et al. 2003; Sarangi et al. 2014). A prior study published in 2009 found that *P. multocida* isolates from chickens, turkeys, and ducks were all genetically distinct from each other. The study also found that the isolates from different poultry species had different surface proteins and virulence factors (Wang et al. 2009). This suggests that *P. multocida* strains that infect different poultry species may be more or less virulent, and that they may respond differently to treatment.

The high degree of variation in avian isolates of *P. multocida* between different poultry species is a major challenge for vaccine development. It is difficult to develop a vaccine that will protect against all strains of the organism in all poultry species.

In particular, pneumonia is a common pathological finding in turkeys with fowl cholera; however, morphological descriptions of *P*. *multocida* are observed (Quinn et al. 2011). Additionally, the histopathological lesions in the infected ducks were found to be more severe than those detected in the infected chickens which were characterized by multiple granulomata in most examined organs. The immunohistochemical (IHC) positive reaction against an antigen of *P. multocida* was more intensely stained and widely distributed in all examined organs of infected ducks than in chickens (Apinda et al. 2020) and so on.

Little is known about the genetic and histopathological characteristics of *P. multocida* isolates circulating in different avian species. The pathognomonic features of *P. multocida* can also change over time due to its ability to acquire new genes through horizontal gene transfer when it encounters other bacteria, such as *E. coli* (Wilson et al. 1993; El-Demerdash et al. 2023a; Megahed et al. 2023). This can lead to the development of new more virulent and difficult to treat strains of *P. multocida* isolates. Multidrug resistance has been exacerbated worldwide, resulting in a public health threat. Several recent studies have reported the emergence of multidrug-resistant bacterial pathogens from different origins necessitating proper use of antibiotics. Routine application of antimicrobial susceptibility testing is necessary to detect the antibiotic of choice and to screen for the emerging MDR strains (El-Demerdash et al. 2018; Algammal et al. 2021, 2022a, 2023; Ebrahem et al. 2023).

Being aware of the pathognomonic features of *P. multocida* isolates is important for the development of new diagnostic tests, vaccines, and treatments for infections caused by this bacterium. This study aims to understand the characteristics and molecular pathogenesis of various *P. multocida* isolates circulating in chickens, ducks, quails, and turkeys providing a comprehensive investigation and epidemiological mapping of the prevalence and diversity of *P. multocida* among various avian species in Sharkia Governorate*.*

## Materials and methods

### Ethical approval and sampling

With approval number ZU-IACUC/2/F/216/2023, the study was carried out with the approval of the Faculty of Veterinary Medicine, Zagazig University, in accordance with its rules. A total of 317 birds (153 chickens, 36 quails, 38 turkeys and100 ducks), apparently healthy, diseased and dead were randomly collected from seven different districts in Sharkia Governorate of different types of production sectors, breeds, and ages during the period between July 2022 and March 2023. Samples of dead birds were collected from the liver, heart, lung, trachea, brain, kidney, and spleen while tracheal swabs were collected from live ones and all samples were subjected to bacterial examination. The sample collection procedures according to Panna et al. (2015) were utilized.

### Pathological examinations

The examinations were conducted on specimens from the tracheas and lungs of infected birds (chickens, ducks, quails, and turkeys) that had either been sacrificed or were freshly dead. The specimens were then fixed in 10% buffered neutral formalin and paraffin sections of 2–3-micron thickness were prepared and stained with hematoxylin and eosin. These sections were then examined microscopically (Suvarna et al. 2013).

### Isolation and identification of *P. multocida*

The isolation procedures were conducted by scorching the surface of the organs with a hot spatula and then sterilizing loopfuls, and inoculating swabs onto Tryptone Soya Broth (TSB, OXOID, Hampshire, United Kingdom). The inoculated TSBs were then incubated aerobically for 24 h at 37 °C and streaked onto 5% sheep blood agar and MacConkey’s agar (OXOID, Hampshire, United Kingdom) for 24 h incubation period at 37 °C. Pure colonies were recognized morphologically using the Gram stain, Leishman's staining method, and biochemical assays (catalase, oxidase, nitrate, methyl red, Voges–Proskauer, sugar fermentation, indole, citrate, gelatin liquefaction and urease) (Carter 1984; Markey et al. 2013)**.**

### Molecular confirmation and typing

These assays were conducted in the Biotechnology Unit, Animal Health Research Institute, Zagazig Branch, Egypt, following a manual previously published in Shalaby et al. (2021).

DNA was extracted from each isolate using the QIAamp DNA Mini kit (Qiagen, Germany, GmbH Catalogue no.51304) and PCR amplification was performed using primers listed in Table 1.

**Table 1:** Oligonucleotide primer sequences used in this study

Target genes	Nucleotide sequence (5′ → 3′)	Amplicon size (bp)	Annealing temperature (^◦^C)	References
Bacterial confrimatin and typing				
*Kmt1*	F: ATCCGCTATTTACCCAGTGG	460	55	Townsend et al. (1998)
	R: GCTGTAAACGAACTCGCCAC			
*hyaD-hyaC* (Serogroup B)	F: TGCCAAAATCGCAGTGAG	1044	55	Townsend et al. (2001)
	R: TTGCCATCATTGTCAGTG			
*BcbD* (Serogroup C)	F: CATTTATCCAAGCTCCACC	760	55	Townsend et al. (2001)
	R: GCCCGAGAGTTTCAATCC			
*DcbF* (Serogroup D)	F: TACAAAAGAAAGACTAGGAGCCC	657	55	Townsend et al. (2001)
	R: CATCTACCCACTCAACCATATCAG			
*EcbJ* (Serogroup E)	F: TCCGCAGAAAATTATTGACTC	511	55	Townsend et al. (2001)
	R: GCTTGCTGCTTGATTTTGTC			
*FcbD* (Serogroup F)	F: AATCGGAGAACGCAGAAATCAG	851	55	Townsend et al. (2001)
	R: TTCCGCCGTCAATTACTCTG			
Virulence genes				
*pfh*A	F: TTCAGAGGGATCAATCTTCG	286	55	Tang et al. (2009)
	R: AACTCCAGT TGGTTTGTCG			
*ptf*A	F: TGTGGAATTCAGCATTTTAGTGTGTC	468	55	Tang et al. (2009)
	R: TCATGAATTCTTATGCGCAAAATCCTGCTGG			
*fim*A	F: CCATCGGATCTAAACGACCTA	866	55	Tang et al. (2009)
	R: AGTATTAGTTCCTGCGGGTG			
*exb*B	F: TTGGCTTGTGATTGAACGC	291	55	Tang et al. (2009)
	R: TGCAGGAATGGCGACTAA A			
*pm*HAS	F: TCAATGTTTGCGATAGTCCGTTAG	430	60	Tang et al. (2009)
	R: TGGCGAATGATCGGTGATAGA			
*tox*A	F: CTTAGATGAGCGACAAGG	864	55	Liu et al. (2017)
	R: GAATGCCACACCTCTATAG			
*omp*A	F: CGCATAGCACTCAAGTTTCTCC	201	60	Tang et al. (2009)
	R: CATAAACAGATTGACCGAAACG			
*omp*H	F: CGCGTATGAAGGTTTAGGT	438	55	Tang et al. (2009)
	R: TTTAGATTGTGCGTAGTCAAC			
*sod*A	F: TACCAGAATTAGGCTACGC	361	60	Vickers (2017)
	R: GAAACGGGTTGCTGCCGCT′			
*sod*C	F: AGTTAGTAGCGGGGTTGGCA	253	60	Vickers (2017)
	R: TGGTGCTGGGTGATCATCATG			
*nan*H	F: CACTGCCTTATAGCCGTATTCC	964	60	Vickers (2017)
	R: AGCACTGTTACCCGAACCC			
*hgb*A	F: TGGCGGATAGTCATCAAG	420	60	Vickers (2017)
	R: CCAAAGAACCACTACCCA			
*oma*87	F: ATGAAAAAACTTTTAATTGCGAGC	984	60	Vickers (2017)
	R: TGACTTGCGCAGTTGCATAAC			
Resistance genes				
*erm*X	F: TCCTTACCAGTGCCCTTATCC	390	65	Rosato et al. (2001)
	R: GAGTTCCAGCGCATCACC			
*dfr*A1	F: CTCACGATAAACAAAGAGTCA	201	50	Abdolmaleki et al. (2019)
	R: CAATCATTGCTTCGTATAACG			
*mcr*1	F: CGGTCAGTCCGTTTGTTC	305	60	Zou et al. (2017)
	R: CTTGGTCGGTCTGTAGGG			
*sul*1	F: CGG CGT GGG CTA CCT GAA CG	433	50	Heuer and Smalla (2007)
	R: GCC GAT CGC GTG AAG TTC CG			
*bla*ROB-1	F: AATAACCCTTGCCCCAATTC	685	60	Klima et al. (2014)
	R: TCGCTTATCAGGTGTGCTTG			
*tet*H	F: ATACTGCTGATCACCGT	1076	60	Klima et al. (2014)
	R: TCCCAATAAGCGACGCT			

### Antimicrobial susceptibility testing

The sensitivity test was performed using the disk diffusion method on Mueller–Hinton Agar (OXOID), according to the procedure recommended by Bauer et al. (1966). All *P. multocida* isolates were validated towards 13 antimicrobial drugs (OXOID) of 11 classes with the following concentrations (in μg/disk): Aminoglycosides (Amikacin AK; 30; Neomycin N; 30), Penicillins (Amoxicillin-clavulanic acid AMC; 30, Ampicillin AMP; 10), Cephalosporins (Cephradine CE; 30), Tetracyclines (Doxycycline DO; 30), Amphenicols (Florfenicol FFC; 30), Sulfonamides (Sulfamethoxazole-trimethoprim SXT; 25), Polymyxin (Colistin CT;10), Macrolides (Erythromycin E; 15), Lincosamide (Lincomycin L; 2), Aminocyclitol (Spectinomycin SH; 25), and Quinolones (Enrofloxacin ENR; 5).

The inhibition zones, in millimeters, were measured in duplicate and scored as sensitive, intermediate, and resistant categories following the critical breakpoints recommended by the Clinical and Laboratory Standards Institute (CLSI 2020). Isolates resistant to ≥ 3 different antimicrobial classes were classified as multidrug-resistant (MDR). The multiple antibiotic resistance (MAR) index for each isolate was calculated as the number of antimicrobials to which the isolate displayed resistance divided by the number of antimicrobials to which the isolate had been tested (Tambekar et al. 2006). The tested isolates were categorized as multidrug-resistant (MDR), extreme drug-resistant (XDR), and pan-drug-resistant (PDR) as described by Magiorakos et al. (2012).

### Detection of virulence and antimicrobial resistance genes

Plasmid DNA was extracted from bacterial isolates using Thermo Scientific GeneJET Plasmid Miniprep Kit (Thermo, Germany, Catalogue no. K0503) for detection of antimicrobial resistant genes.

PCR amplification of virulence-related genes of *P. multocida* as well as antimicrobial resistance genes such as *erm(*X*)* (macrolide, lincosamides and streptogramins resistance), *sul1*(sulfonamide resistance), *tet(*H*)* (tetracycline resistance), *bla* ROB-1 (beta-lactam resistance), *mcr*1(colistin resistance), and *dfr*A1 (trimethoprim resistance) were performed by PCR assays using the oligonucleotide primer sequences presented in Table 1.

A PTC-100 programmable thermal cycler (Peltier-Effect cycling, MJ, UK) was used to conduct the PCR assays. The reaction mixture’s final volume was adjusted to 25 μL and comprised 12.5 μL of DreamTaq TM Green Master Mix (2X) (Fermentas, USA), 0.4 μL of each primer at 100 pmoL (Sigma, USA), 5 μL of template DNA, and 25 μL of nuclease-free water. The cycling conditions were: 30 cycles; 95 °C for 45 s, primer annealing (TA, Table 1) for 45 s, and 72 °C for 45 s.

On a 1.5% agarose gel (Applichem, Germany, GmbH), the PCR products were separated by electrophoresis, and the gel was photographed using a gel documentation framework (Alpha Innotech, Biometra). The data was analyzed by computer software. As a quality control, *P. multocida* ATCC® 43137™ was utilized.

### Statistics and data analyses

Chi-square or Fisher’s exact test (as needed) were used to compare categorical variables and two-tailed, unpaired student t-test was used to compare numerical variables. In all statistics, *p*-value at 0.05 were used as cutoff level for significance. The statistics were done using base functions in the R software version 4.3.1.

Heat maps and hierarchical clustering was used to visualize the overall occurrence of analyzed traits in the isolates and the relation among them and was done using the Pheatmap package in r software version 4.3.1 (Kolde 2012). Before correlation analyses, variables were tested for normality using Q–Q plot. Pearson correlation was estimated and visualized using the Hmisc package in R software version 4.3.1 (Martins 2022). Significant correlation pairs were determined at ***p***-value of 0.05. The frequencies of studied genes/phenotypes were plotted as stacked bar graph using ggplot package in R software version 4.3.1 (Wickham et al. 2009).

To determine the influence of various predictor variables on the infection outcome, we treated each avian species (e.g. chicken) as a stand-alone dataset. For each species, we run a univariate logistic regression model on the independent variables to select significant ones as they relate to infection outcome, then a backward selection of those significant was applied to run a multivariate logistic regression model. Since the effect of some predictors on the infection outcome varies according to other variables, we included an interaction term in the regression equation. These analyses yielded β-coefficient, odds-ratio, and ***p***-value for each predictor. The analyses were done using glm function in R software version 4.3.1.

### Epidemiological mapping for pasteurellosis infection among examined avian species

The final collected data were subjected to ArcGIS application for geo-mapping the rate of *P. multocida* infection through Sharkia governorate, Egypt.

## Results

### Clinical signs

General respiratory disorders were observed in most of the examined infected birds. In turkeys, a mild swelling of the head with eye discharge was observed in some cases. Sneezing, depression, mucoid discharge from the mouth, ruffled feathers, increased respiratory rate, and diarrhea which was most common in chickens and quail, were observed. Ducks suffered from tracheal rales with extended neck and nasal discharge. High morbidity rates (42–86%) were detected, while the mortality rate was mild to moderate in the inspected farms (5–31%). Decreased feed intake and weight loss were detected with the progress of the disease, especially in cases of severe infection*.*

### Postmortem examination

The prevalence of gross lesions based on the trachea and lung were the most affected organs exhibiting inflammation in variable severity degrees. Congestion was marked especially in ducks, and catarrhal tracheitis represented in the trachea was filled with yellowish exudates in other cases such as in quails and chickens. Mucosal hemorrhagic spots may appear in severe cases. Examined lungs from positive infected cases appeared in different forms, an edematous, firm with cut surface yielded blood-tinged exudate and mild to moderate hemorrhage of mostly focal distribution, focal to diffuse congestion. The incidence and prevalence of gross lesions based on the examined birds were summarized in the lesion score (Table 2).

**Table 2:** Lesions score of respiratory disorders in trachea of infected birds

Affected tissue	Lesion	Infected bird	Severity
Trachea	Congestion	Quail	+
		Chicken	+
		Duck	+ + +
		Turkey	+ +
	Detached cilia Partial (p) Complete (c)	Quail	+
		Chicken	+
		Duck	+ +
		Turkey	+
	Hemorrhage	Quail	+ +
		Chicken	+
		Duck	+ +
		Turkey	+ +
	Degeneration of tracheal glands	Quail	+
		Chicken	−
		Duck	+ +
		Turkey	+
	Necrosis of chondrocytes	Quail	−
		Chicken	+
		Duck	+ +
		Turkey	−
	Endotheliosis	Quail	+ +
		Chicken	+ +
		Duck	+ + +
		Turkey	−
	Perivascular fibrosis	Quail	+
		Chicken	+ +
		Duck	+ +
		Turkey	−
	Perivascular edema	Quail	+
		Chicken	+ +
		Duck	+ + +
		Turkey	−

### Microscopical findings

**Fig. 1:**
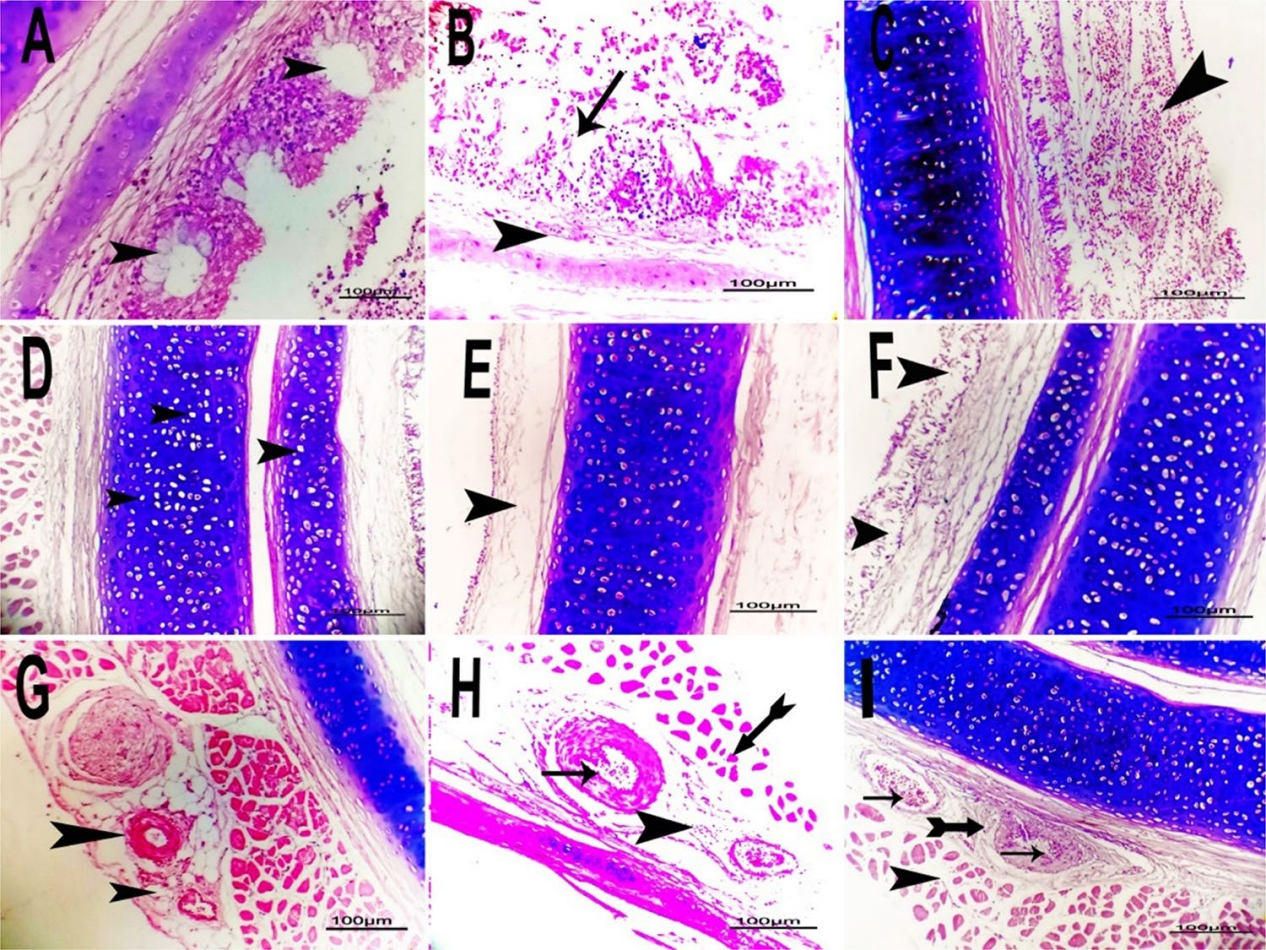
Photomicrograph of H&E-stained sections of *P. multocida* infected tracheas in different examined birds revealed **A**: Cystic dilation of some mucosal glands (arrowhead) in quail. **B**: Detached cilia with haemorrhage (arrow) and cellular infiltration (arrowhead) in Chicken. **C**: Destructed mucosa with severe extravasated erythrocytes (haemorrhage) (arrowhead) in Turkey. **D**: Necrosis of some chondrocytes of tracheal cartilage (arrowhead) in Duck. **E**: Complete destruction of mucosa and tracheal glands (arrowhead) with normal cartilage in Duck. **F**: Partial destruction of mucosa and submucosal glands (arrows head) with normal cartilage (arrow) in Turkey.** G**: Perivascular cellular infiltration and oedema (arrows head) with congestion in Chicken. **H**: Perivascular extravasated erythrocytes (arrowhead) with congestion, endotheliosis (arrow), and atrophy of tracheal muscle fibers (tailed arrow) in Quail. **I**: Congestion of blood vessels, endotheliosis (arrows) with perivascular fibrosis (tailed arrow), and atrophy of tracheal muscle fibers (arrowhead) in Chicken (scale bar = 100 µm)

Tracheal lesions were cystic dilation of some mucosal glands (Fig. 1A), partial to complete detached cilia with hemorrhage and cellular infiltration (Fig. 1B**),** destructed mucosa with severe extravasated erythrocytes (hemorrhage) (Fig. 1C), necrosis of some chondrocytes of tracheal cartilage (Fig. 1D), and complete and partial destruction of mucosa and tracheal glands with or without normal cartilage (Fig. 1E&1F), perivascular cellular infiltration and edema with congestion (Fig. 1G). Perivascular extravasated erythrocytes with vascular congestion, endotheliosis (endothelial cell degeneration), and atrophy of tracheal muscle fibers (Fig. 1H**)** were also observed, along with perivascular fibrosis with or without atrophy of tracheal muscle fibers **(**Fig. 1I**)**.

**Fig. 2:**
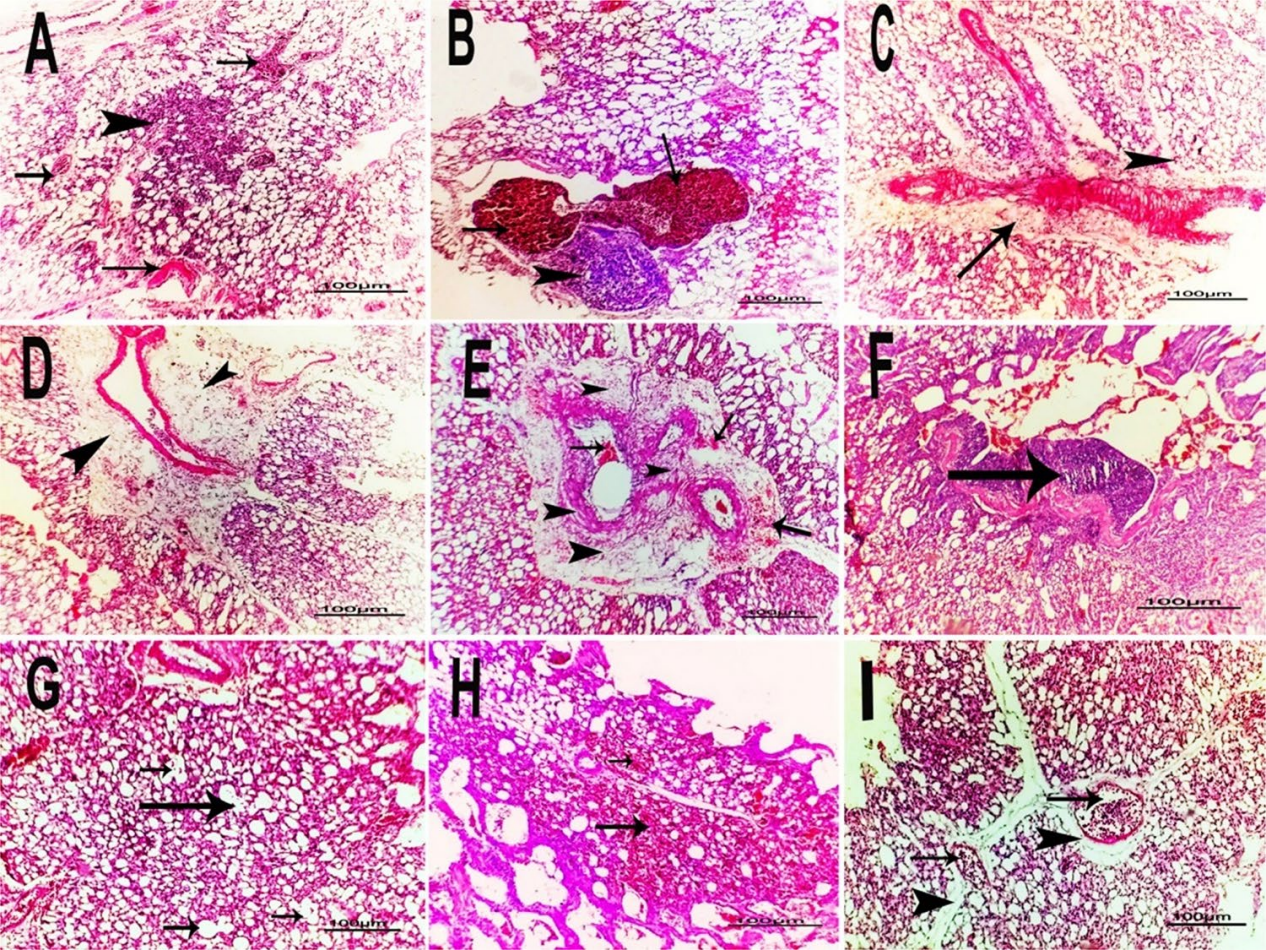
Photomicrograph of H&E-stained sections of *P. multocida* infected lungs in different examined birds revealed **A**: Pneumonia represented in diffuse congestion (arrows) and cellular infiltration (arrowhead) in Turkey. **B**: Severe focal hemorrhage (arrows) with focal cellular infiltration (arrowhead) in Duck.** C**: Perivascular cellular infiltration (arrow) with focal partial alveolar stenosis (arrowhead) in Quail. **D**: Perivascular oedema, fibrosis, and cellular infiltration (arrows head) in Chicken. **E**: Perivascular fibrosis (arrowhead) with extravasated erythrocytes in Duck. **F**: Hyperplasia of the bronchial epithelium (arrow) in Turkey. **G**: Partial focal emphysema (arrows) in Chicken. **H**: Interstitial extravasated erythrocytes (arrows) in Duck. **I**: Congestion of blood vessels (arrows) with perivascular edema (arrows head) in Quail (scale bar = 100 µm)

**Fig. 3:**
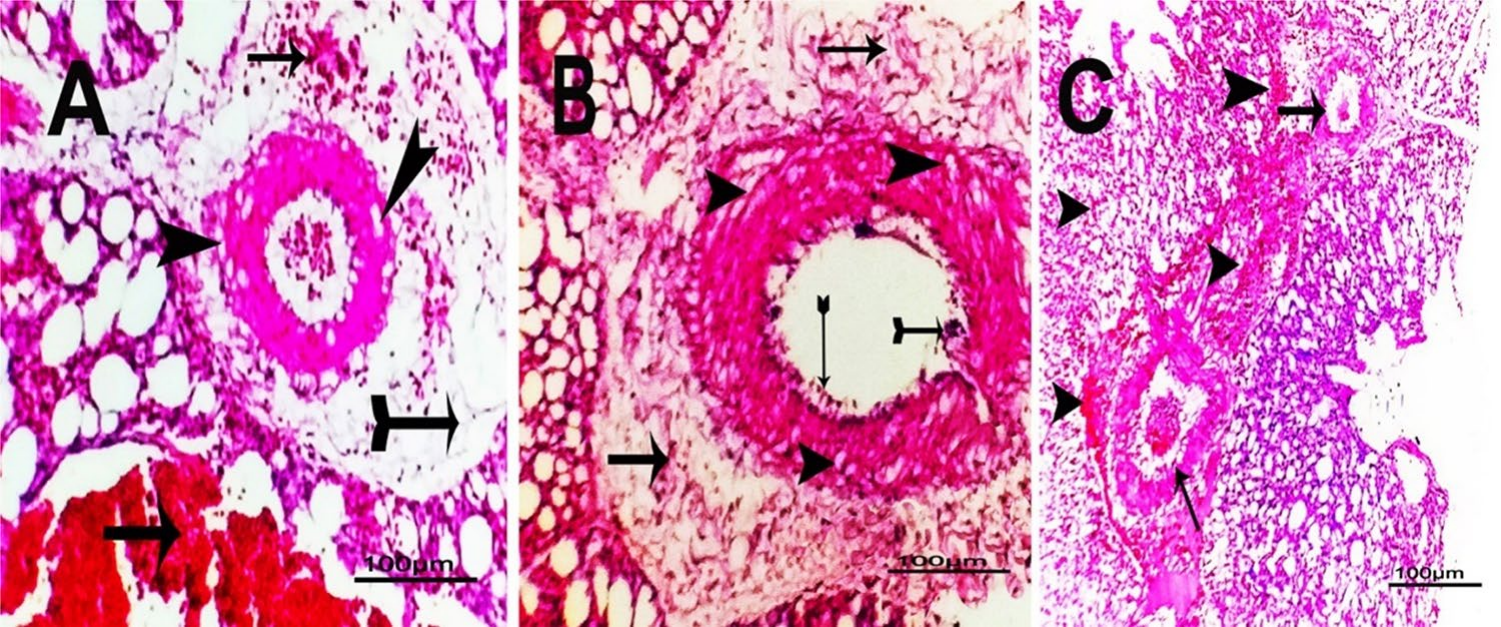
Photomicrograph of H&E-staine-d sections of *P. multocida* infected lungs in different examined birds revealed **A**: Vacuolation of vascular tunica media (arrowhead) with perivascular edema (tailed arrow) and cellular infiltration (arrows) in Chicken. **B**: Endotheliosis (tailed arrows) with vacuolation of vascular tunica media (arrowhead) and perivascular fibrosis(arrows) in Turkey.** C**: Perivascular cellular infiltration (arrowhead) with endotheliosis (arrows) in Quail (scale bar = 100 µm)

Lung lesions were more prominent, especially in ducks and included pneumonia represented in diffuse congestion and cellular infiltration with variable degrees of severity (Fig. 2A), bronchopneumonia, mild to severe focal to diffuse interstitial hemorrhage with focal inflammatory cellular infiltration **(**Fig. 2B**)** of mostly neutrophile, macrophages, and perivascular cellular infiltration with focal partial alveolar stenosis (Fig. 2C). Perivascular oedema, and fibrosis with or without cellular infiltration (Fig. 2D) were also observed, along with perivascular fibrosis with extravasated erythrocytes (Fig. 2E), hyperplasia of bronchial epithelium accompanied bronchopneumonia in some cases (Fig. 2F) and partial focal emphysema only in some chronic cases (Fig. 2G). Interstitial extravasated erythrocytes (Fig. 2H) and congestion of blood vessels with perivascular oedema (Fig. 2I) were also detected. Vascular changes in the lung were common and more observed in most cases which were represented by vacuolation of vascular tunica media with perivascular edema and cellular infiltration (Fig. 3A), endotheliosis and perivascular fibrosis (Fig. 3B) and perivascular cellular infiltration (Fig. 3C).

Full coverage of all previously mentioned microscopical lesions in different examined birds was illustrated in Table 3.

**Table 3:** Lesions score of respiratory disorders in lung of infected birds

Affected tissue	Lesion	Severity	Infected bird
Lung	Pneumonia	Quail	+ +
		Chicken	+ + +
		Duck	+ + +
		Turkey	+ +
	Alveolar stenosis	Quail	+ +
		Chicken	+
		Duck	+ +
		Turkey	+
	Hemorrhage	Quail	+ +
		Chicken	+ +
		Duck	+ + +
		Turkey	+ + +
	Emphysema	Quail	−
		Chicken	+ +
		Duck	+ +
		Turkey	+
	Hyperplasia of bronchial epithelium	Quail	−
		Chicken	−
		Duck	+ + +
		Turkey	+ +
	Vascular changes	Quail	+ +
		Chicken	+ + +
		Duck	+ + +
		Turkey	+
	Perivascular fibrosis	Quail	+
		Chicken	+ +
		Duck	+ + +
		Turkey	+ +
	Perivascular edema	Quail	+
		Chicken	+ +
		Duck	+ + +
		Turkey	+

### The phenotypic characteristics of the recovered *P. multocida* isolate

The isolates were examined microscopically, they were Gram-negative bipolar coccobacilli, dew grey, drop-like mucoid, non-hemolytic on blood agar, and failed to develop on MacConkey agar. The recovered isolates tested positive for catalase, oxidase, indole production, nitrate reduction, d-glucose, d-mannitol, galactose, fructose, and sucrose fermentation, and negative for methyl red, Voges-Proskauer, urease, citrate utilization, and gelatin liquefaction.

### The prevalence of *P. multocida* in various birds and breeds and relation to the locality, breeding type, and sampled organs

From 317 tested birds, a total of 30 (9.4%) *P. multocida* isolates were obtained; all of these were of capsular type A apart from one quail isolate of type D. The climate changes, breed, age, location, clinical symptoms, and sample type had impacts on the incidence rate of *P. multocida* in the hosts. Breed was the most significant factor in chickens, while age and breed were less important in other species (Tables 4 and 5).

**Table 4:** Univariate logistic regression model for each bird species

	B-coefficient	Odds ratio	Low	High	*p*-value	Significance
Chicken						
District						
(Intercept)	− 2.0	0.1	− 3.9	− 0.7	0.0	*
Abo kbir	− 0.4	0.7	− 2.3	1.7	0.7	Ns
Belbis	− 15.6	0.0	#N/A	97.2	1.0	Ns
Dereb negm	− 0.2	0.8	− 3.3	2.3	0.9	Ns
El- Hessneia	0.0	1.0	− 1.9	2.1	1.0	Ns
Fakous	− 0.1	0.9	− 3.2	2.4	1.0	Ns
Menia El-Kamh	− 0.4	0.7	− 2.1	1.6	0.7	Ns
Breed						
(Intercept)	− 1.8	0.2	− 2.8	− 1.1	0.00	*
Breed: Broiler	− 0.1	0.9	− 1.3	1.1	0.84	Ns
Breed: Layer	− 2.1	0.1	− 5.0	− 0.3	0.05	*
Age						
(Intercept)	− 0.8	0.4	− 3.7	2.1	0.58	Ns
Age	− 0.2	0.9	− 0.5	0.1	0.32	Ns
Breeding system						
(Intercept)	− 1.6	0.2	− 2.4	− 0.9	0.00	*
Farm	− 1.2	0.3	− 2.4	− 0.1	0.04	*
Duck						
District						
(Intercept)	− 19.6	0.0	#N/A	480.1	1.00	Ns
Abo kbir	16.9	20,957,737.3	− 981.5	#N/A	1.00	Ns
Belbis	17.5	41,004,268.6	− 429.9	#N/A	1.00	Ns
Dereb negm	0.0	1.0	− 694.1	865.0	1.00	Ns
El-Hessneia	0.0	1.0	− 293.0	273.5	1.00	ns
Fakous	19.6	314,366,059.1	− 427.9	#N/A	1.00	ns
Menia El-Kamh	16.2	10,478,868.6	− 431.3	#N/A	1.00	ns
Breed						
(Intercept)	− 1.3	0.3	− 2.8	− 0.1	0.05	*
Molar	− 0.4	0.7	− 2.6	1.6	0.69	ns
Muscovy	− 2.0	0.1	− 4.1	− 0.1	0.04	*
Pekin	− 18.3	0.0	#N/A	232.5	0.99	Ns
Age						
(Intercept)	− 1.9	0.2	− 4.8	0.8	0.19	Ns
Age	− 0.1	0.9	− 0.4	0.2	0.61	Ns
Breeding system						
(Intercept)	− 0.2	0.8	− 1.4	1.0	0.76	Ns
Farm	− 3.6	0.0	− 5.7	− 1.9	0.00	*
Turkey						
Breeding: system						
(Intercept)	0.7	2.0	− 1.7	3.8	0.57	Ns
Farm	− 3.5	0.0	− 6.9	− 0.8	0.01	*
Age						
(Intercept)	1.5	4.4	-5.3	8.1	0.65	Ns
Age	− 0.4	0.7	-1.1	0.3	0.27	Ns
Quails						
Age						
(Intercept)	14.6	2,255,294.5	4.8	30.6	0.02	*
Age	− 3.0	0.1	− 6.0	− 1.2	0.01	*
Breeding: system						
(Intercept)	− 2.0	0.1	− 3.9	− 0.7	0.01	*
Farm	0.3	1.4	− 1.6	2.5	0.73	Ns
District						
(Intercept)	− 1.5	0.2	− 2.6	− 0.6	0.00	**
Belbis	− 17.0	0.0	#N/A	230.7	0.99	Ns

* Significant, Ns: Non- significant

**Table 5:** Multivariate logistic regression with interaction terms per species

	B-coefficient	Odds ratio	Low	High	*p-*value	Significance
Chicken						
District						
(Intercept)	1.37	3.94	− 1.46	4.97	0.37	Ns
Abo kbir	− 0.90	0.40	− 3.46	1.64	0.46	Ns
Belbis	− 19.55	0.00	#N/A	220.09	1.00	Ns
Dereb negm	0.90	2.45	− 2.53	3.93	0.55	Ns
El- Hessneia	0.39	1.48	− 2.04	3.07	0.76	Ns
Fakous	− 3.19	0.04	− 7.44	0.12	0.08	Ns
Menia El-Kamh	0.57	1.77	− 1.72	3.23	0.64	Ns
Breed						
Broiler	− 2.49	0.08	− 5.87	0.15	0.08	Ns
Layer	− 4.35	0.01	− 8.44	− 1.15	0.01	*
Interactions						
Farm	− 3.97	0.02	− 7.60	− 1.01	0.01	*
Broiler: farm	2.71	15.01	− 0.86	6.74	0.15	Ns
Layer: farm	− 13.04	0.00	− 529.66	71.42	0.99	Ns
Duck						
(Intercept)	− 0.41	0.67	− 2.43	1.39	0.66	Ns
Molar	20.97	1,281,803,142.55	− 1924.02	#N/A	1.00	Ns
Muscovy	20.97	1,281,803,185.67	− 3581.41	#N/A	1.00	Ns
Pekin	− 20.16	0.00	#N/A	1237.46	1.00	Ns
Interactions						
Farm	− 1.67	0.19	− 4.97	0.98	0.23	Ns
Molar: farm	− 39.46	0.00	#N/A	1208.49	1.00	Ns
Muscovy: farm	− 22.90	0.00	#N/A	3220.25	1.00	Ns
Pekin: farm	1.67	5.33	#N/A	1353.40	1.00	Ns
Quails						
(Intercept)	13.24	562,738.71	2.87	29.78	0.04	*
Belbeis	− 16.31	0.00	#N/A	449.64	1.00	Ns
Age	− 2.69	0.07	− 5.81	− 0.86	0.02	*
Farm	0.09	1.10	− 3.18	2.93	0.95	Ns

*Ns* non-significant

* Significant

In chicken and ducks, the most susceptible breed was Baladi with an average age of 8–9 weeks; diseased samples recorded a higher prevalence than dead birds.

In quails, age as the most affectable factor with a range of 4–5 weeks. In turkeys, the type of sample and age were the influential factors; the higher prevalence was recorded in dead samples with an average age of 9 weeks.

### Antimicrobial susceptibility patterns and genotypic profiles of *Pasteurella multocida* isolates

The majority of isolates exhibited high levels of resistance to amoxicillin-clavulanic acid (86.6%), erythromycin (73.3%), and colistin (60%).

All quail isolates showed absolute resistance to amoxicillin-clavulanic acid, ampicillin, colistin, sulfamethoxazole-trimethoprim, and erythromycin. However, all turkey isolates were susceptible to ampicillin and sulfamethoxazole-trimethoprim. The average MAR index of all *P. multocida* was 0.43, ranging from 0.23 to 0.77. The highest MAR index (0.77) was found in an isolate recovered from a duck which represent XDR pattern of resistance (Table 6).

Florfenicol and enrofloxacin were the drugs of choice for all the obtained avian isolates.

**Table 6:** Source, capsular type, antimicrobial resistance profiles, virulence, and resistance genes of *P. multocida* isolates recovered from different examined avian species

Isolate	Capsular type	Source	Resistance Profiles*	MAR Index^c^	Resistance to antimicrobials (n = 11)	Virulence genes	Resistance genes
1P	A	Chicken	AMC, DO, E, AMP, CE	0.38	4 MDR^a^	*pfh*A, *ptf*A, *fim*A, *exb*B, *pm*HAS, *omp*A, *omp*H, *sod*A, *sod*C, *hgb*A, *oma*87	*erm*X
2P	A	Chicken	AMC, SXT, CT, E, AMP, L, CE	0.54	6 MDR^a^	*pfh*A, *ptf*A, *fim*A, *exb*B, *pm*HAS, *omp*A, *omp*H, *sod*A, *sod*C, *hgb*A, *oma*87	*erm*X, *mcr-*1
3P	A	Chicken	AMC, DO, SXT, CT, E, AMP, L	0.54	6 MDR^a^	*ptf*A, *fim*A, *exb*B, *pm*HAS, *omp*H, *sod*A, *sod*C, *hgb*A, *oma*87	*erm*X, *mcr-*1, *sul*-1
4P	A	Chicken	AMC, SXT, CT, E, AMP, L, CE	0.54	6 MDR^a^	*ptf*A, *exb*B, *pm*HAS, *sod*A, *sod*C, *hgb*A, *oma*87	*erm*X, *dfr*A1, *bla*ROB-1
5P	A	Duck	AMC, SXT, CT, E, AMP, L	0.46	5 MDR^a^	*pfh*A, *ptf*A, *exb*B, *pm*HAS, *omp*A, *omp*H, *sod*A, *sod*C, *hgb*A, *oma*87, *nan*H	*erm*X, *bla*ROB-1, *mcr-*1, *sul*-1
81P	A	Chicken	DO, L, CE	0.23	3 MDR^a^	*pfh*A, *ptf*A, *exb*B, *pm*HAS, *omp*A, *omp*H, *sod*A, *sod*C, *hgb*A, *oma*87, *nan*H	*tet*H
82P	A	Chicken	AMC, DO, L, CE	0.31	4 MDR^a^	*ptf*A, *exb*B, *pm*HAS, *omp*H, *sod*A, *sod*C, *hgb*A, *oma*87, *nan*H, *fim*A,	*tet*H
83P	A	Duck	DO, E, CT, CE	0.31	4 MDR^a^	*pfh*A, *ptf*A, *fim*A, *pm*HAS, *sod*A, *sod*C, *nan*H, *oma*87	*tet*H
84P	A	Chicken	AMC, DO, AMP, N, CE	0.38	4 MDR^a^	*pfh*A, *ptf*A, *fim*A, *pm*HAS, *omp*A, *sod*A, *sod*C, *oma*87	*tet*H
131P	A	Chicken	AK, AMC, DO, SXT, E, CE	0.46	6 MDR^a^	*ptf*A, *exb*B, *omp*A, *omp*H, *sod*A, *sod*C, *hgb*A, *oma*87, *nan*H	*erm*X, *dfr*A1, *sul*-1
132P	A	Chicken	SXT, E, CT, CE	0.31	4 MDR^a^	*pfh*A, *ptf*A, *exb*B, *pm*HAS, *omp*H, *sod*A, *sod*C, *hgb*A, *oma*87	*mcr-*1, *sul*-1
133P	A	Chicken	SXT, E, SH	0.23	3 MDR^a^	*ptf*A, *pm*HAS, *sod*A, *sod*C, *oma*87	*erm*X, *sul*-1
171P	A	Chicken	AMC, DO, SXT, E, AMP, SH	0.46	5 MDR^a^	*ptf*A, *exb*B, *pm*HAS, *omp*A, *tox*A *omp*H, *sod*A, *hgb*A, *oma*87	*erm*X, *mcr-*1, *sul*-1, *tet*H
172P	A	Chicken	AMC, SXT, CE	0.23	3 MDR^a^	*pfh*A, *ptf*A, *fim*A, *pm*HAS, *omp*H, *sod*A, *hgb*A, *oma*87	*bla*ROB-1, *dfr*A1, *sul*-1
191P	A	Duck	AK, AMC, DO, SXT, E, CT, AMP, N, SH	0.69	7 MDR^a^	*pfh*A, *ptf*A, *fim*A, *omp*A, *tox*A, *omp*H, *sod*A, *sod*C, *oma*87, *nan*H	*erm*X, *bla*ROB-1, *mcr-*1, *sul*-1, *dfr*A1
192P	A	Duck	AMC, SXT, CT, CE, SH	0.38	5 MDR^a^	*ptf*A, *exb*B, *pm*HAS, *omp*H, *sod*A, *sod*C, *hgb*A, *oma*87, *nan*H	*bla*ROB-1, *mcr-*1, *sul*-1, *dfr*A1
195P	A	Duck	AK, AMC, DO, SXT, E, CT, AMP, L, CE, SH	0.77	9 XDR^b^	*pfh*A, *ptf*A, *fim*A, *exb*B, *omp*H, *sod*A, *sod*C, *oma*87, *nan*H	*erm*X, *bla*ROB-1, *mcr-*1
202P	A	Duck	AK, AMC, SXT, E, CT, AMP	0.46	5 MDR^a^	*pfh*A, *ptf*A, *fim*A, *exb*B, *omp*H, *sod*A, *sod*C, *oma*87, *nan*H, *pm*HAS	*bla*ROB-1, *tet*H
210P	A	Chicken	AMC, SXT, CE, SH	0.31	4 MDR^a^	*ptf*A, *exb*B, *omp*H, *sod*A, *sod*C, *oma*87, *nan*H, *pm*HAS, *hgb*A	*bla*ROB-1
211P	A	Chicken	AMC, DO, SXT, E, CT, AMP, L	0.54	6 MDR^a^	*ptf*A, *fim*A, *sod*A, *sod*C, *oma*87, *nan*H, *hgb*A	*erm*X, *bla*ROB-1, *mcr-*1, *dfr*A1
221P	A	Quail	AMC, DO, E, AMP, CE	0.38	4 MDR^a^	*pfh*A, *ptf*A, *fim*A, *exb*B, *pm*HAS, *omp*A, *omp*H, *sod*A, *sod*C, *oma*87, *nan*H	*erm*X, *bla*ROB-1
222P	A	Quail	AMC, SXT, E, CT, AMP, L, CE	0.54	6 MDR^a^	*pfh*A, *ptf*A, *exb*B, *pm*HAS, *omp*A, *omp*H, *sod*A, *sod*C, *oma*87, *nan*H	*bla*ROB-1
223P	A	Quail	AMC, DO, SXT, E, CT, AMP, L	0.54	6 MDR^a^	*ptf*A, *fim*A, *exb*B, *pm*HAS, *omp*A, *omp*H, *sod*A, *sod*C, *oma*87, *nan*H	*erm*X, *bla*ROB-1, *mcr-*1, *sul*-1, *dfr*A1
224P	D	Quail	AK, AMC, DO, SXT, E, CT, AMP, N, L, CE	0.69	7 MDR^a^	*ptf*A, *omp*A, *tox*A, *omp*H, *sod*A, *sod*C, *oma*87, *nan*H, *hgb*A	*erm*X, *bla*ROB-1, *mcr-*1, *sul*-1, *dfr*A1
225P	A	Quail	AMC, DO, SXT, E, CT, AMP, CE	0.46	5 MDR^a^	*ptf*A, *fim*A, *sod*A, *sod*C, *oma*87	*erm*X, *bla*ROB-1, *dfr*A1
247P	A	Duck	AMC, DO, E, CT, L	0.31	4 MDR^a^	*ptf*A, *fim*A, *exb*B, *pm*HAS, *omp*A, *omp*H, *sod*A, *sod*C, *oma*87, *hgb*A	*erm*X
263P	A	Turkey	AMC, CT, L	0.23	3 MDR^a^	*ptf*A, *fim*A, *exb*B, *pfh*A,*omp*A, *omp*H, *sod*A, *sod*C, *oma*87, *hgb*A	*mcr-*1, *dfr*A1
264P	A	Turkey	AMC, DO, E, CT, L, CE, SH	0.54	7 MDR^a^	*ptf*A, *exb*B, *omp*H, *sod*A, *sod*C, *oma*87, *hgb*A	*erm*X
265P	A	Turkey	AMC, E, CT, L, CE	0.38	5 MDR^a^	*ptf*A, *fim*A, *pfh*A, *sod*A, *sod*C, *oma*87, *hgb*A	*erm*X
273P	A	Turkey	AMC, DO, L, CE	0.31	4 MDR^a^	*ptf*A, *exb*B, *pm*HAS, *omp*H, *sod*A, *sod*C, *oma*87, *hgb*A	*bla*ROB-1, *tet*H

*AK* amikacin; *N* neomycin; *AMC* amoxicillin-clavulanic acid, *AMP* ampicillin, *CE* cephradine; *DO* doxycycline; *FFC* florfenicol; *SXT* sulfamethoxazole-trimethoprim; *CT* colistin, *E* erythromycin; *L* lincomycin; *SH* spectinomycin and *ENR* enrofloxacin

^a^The isolates were resistant to ≥ 1 agent in ≥ 3 antimicrobial categories

^b^The isolates were resistant to ≥ 1 agent in all except ≤ 2 antimicrobial categories

^c^Multiple antibiotic resistance index (average MAR index = 0.43)

**Fig. 4:**
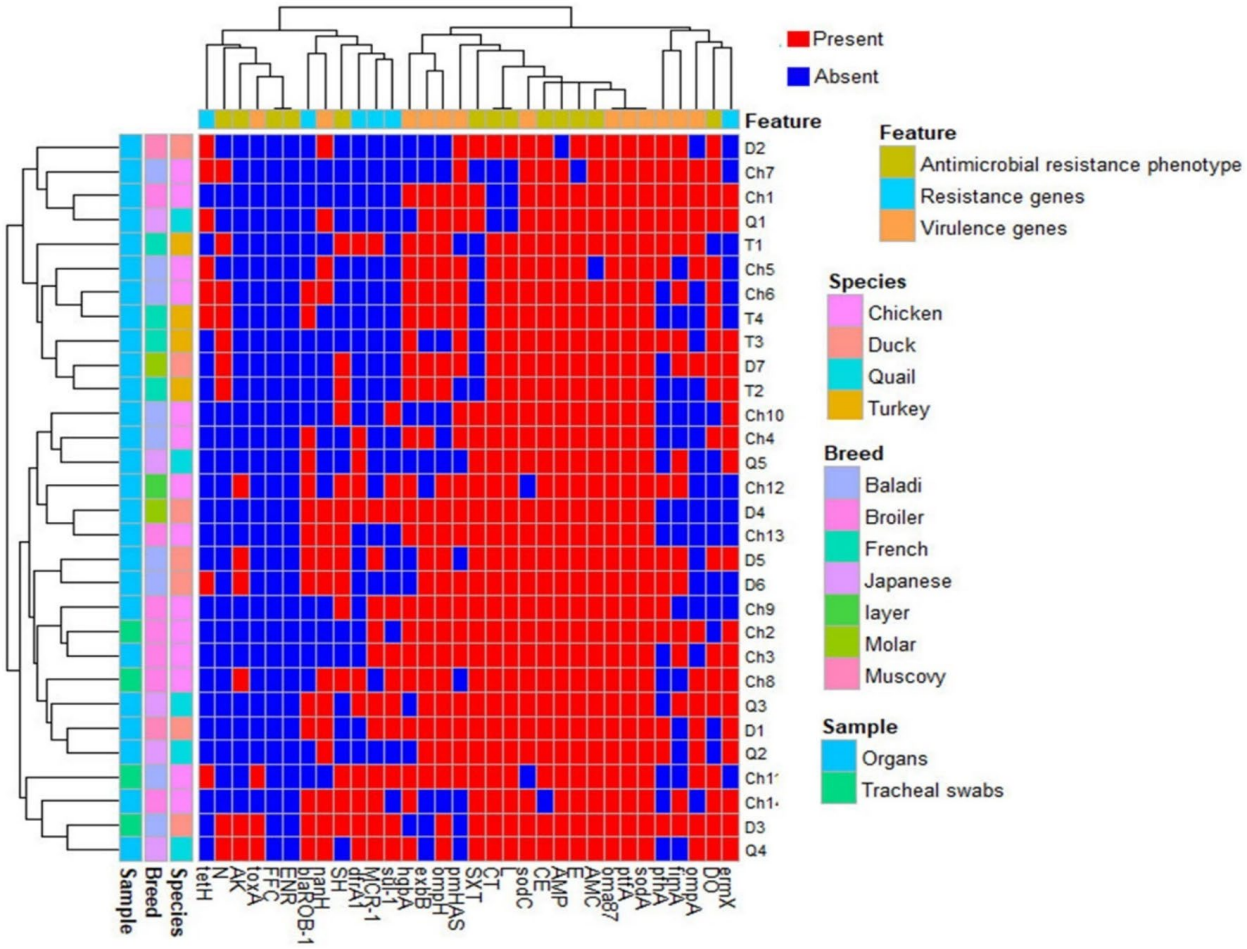
Heatmap supported by dendrogram showing the overview of the distribution of antimicrobial resistance phenotype, genes and virulence genes in the studied bird species. Different bird species, breeds and sample types are shown as color-coded. Red and blue colours indicate presence and absence of respective feature. Dendrogram shows the clustering pattern of the isolates

The association of various isolates is shown in Fig. 4. The analyses of antimicrobial resistance and virulence features revealed that none of the isolates were identical in their profile. They fell into two big clusters; a bigger one with 19 isolates and a smaller one with 11 isolates. Each of these clusters was composed of isolates from different animals and breeds. The small cluster contained isolates only from bird organs, whereas the big cluster was formed from isolates from both organs and tracheal swabs.

There was no particular clustering of analyzed genes or phenotypes. Moreover, the heatmap shows that Baladi chickens are more likely to have resistance to multiple antimicrobials, including ampicillin, amoxicillin-clavulanic acid, cephradine, doxycycline, erythromycin, colistin, and sulfamethoxazole-trimethoprim. These isolates are also more likely to have virulence genes, such as those that encode for adhesins, protections, and enzymes that help the bacteria to evade the host's immune system.

**Fig. 5:**
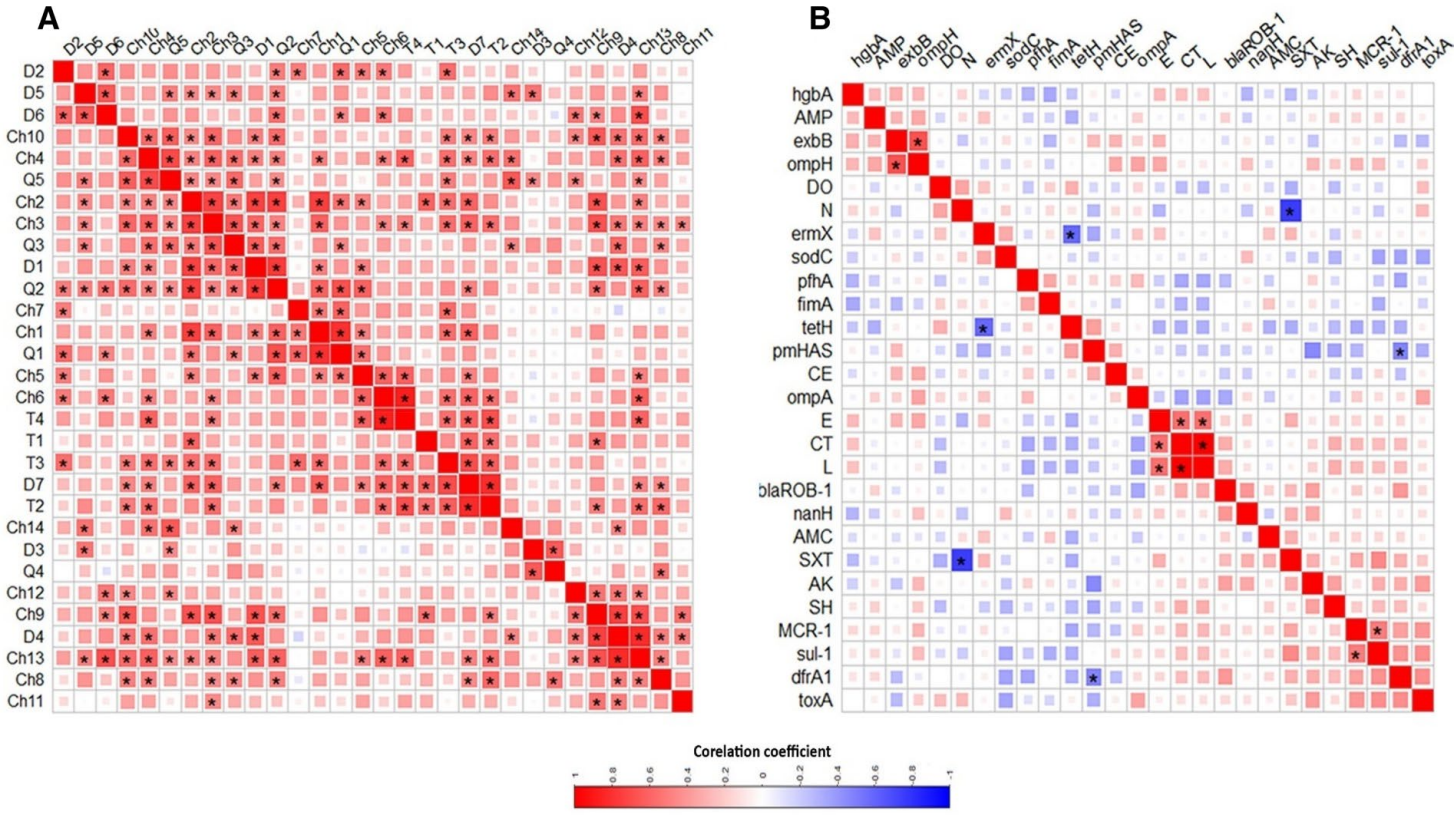
Pairwise correlation of different *Pasteurella* isolates (**A**) and analysed features (**B**). The correlation coefficients are shown as colours on the scale (positive: red, negative: blue). The more intense the colour, the more the stronger the positive or negative correlation. Stars refer to the respective significant correlation (details of the p-value are shown in Table S1)

As shown in Fig. 5 and Table S1, we identified a high positive significant correlation between lincomycin and colistin-resistant phenotypes (R = 1, ***p***-value < 0.0001) as well as a significant moderate positive correlation between colistin and erythromycin (R = 0.6, ***p-***value = 0.001). On the other side, neomycin and sulfamethoxazole-trimethoprim correlated significantly and negatively (R = − 0.8, ***p***-value < 0.0001). Some antimicrobial resistance genes correlated moderately and significantly negatively (for instance *tetH* and *ermX*; R = − 0.6, ***p***-value = 0.0006). Overall, a general negative correlation between antimicrobial resistance and virulence genes was observed with both *dfrA1* and *pmHAS* correlating moderately negatively (R = − 0.5, ***p-***value = 0.007). The ***p***-values in Table S1 show that the correlations are statistically significant meaning that the correlations are not due to chance and are likely to be real.

**Fig. 6:**
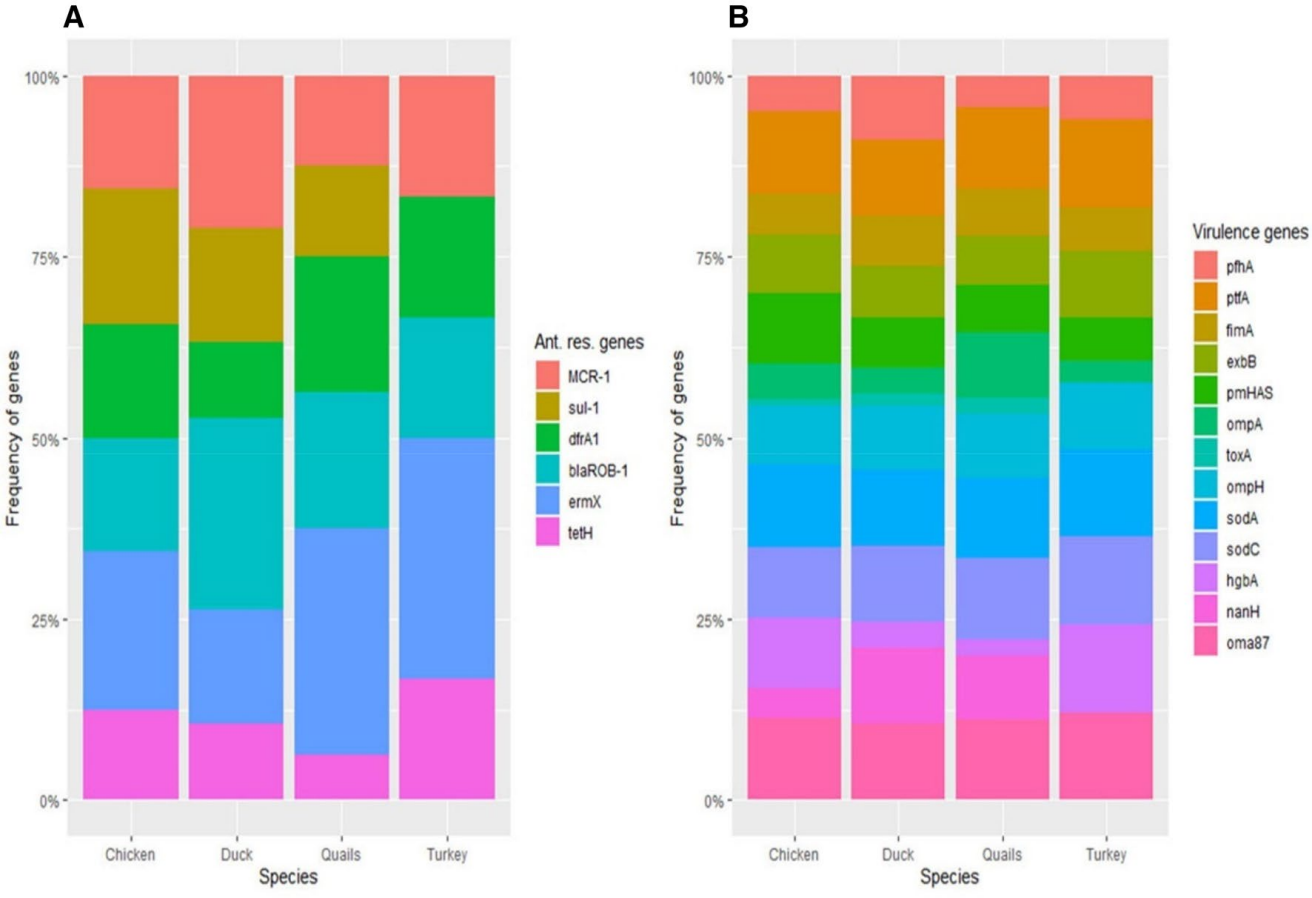
Stacked bar graph showing the frequency of occurrence of both antimicrobial resistance (**A**) and virulence (**B**) genes. Each colour refers to one gene that are color-coded. The source of the isolates is shown on the X-axis

The graph in Fig. 6 shows that the frequency of occurrences of *sodA, ompH, ermX,* and *blaROB-1* genes are the most prevalent, especially in quails compared to other avian species. Also, Table 7 shows that the frequency of antimicrobial resistance and virulence genes is higher in ducks than in chickens, quails, and turkeys. This suggests that ducks are more likely to be exposed to antimicrobial-resistant and virulent bacteria than other species.

**Table 7:** Frequency of antimicrobial resistance phenotypes, genotypes and virulence genes in the studied birds

Variable	Chicken		Duck		Quails		Turkey		X2	*p-*value^1^
	Present	Absent	Present	Absent	Present	Absent	Present	Absent		
AK	2	12	3	3	1	4	0	4	4.5	0.2
AMC	13	1	6	0	5	0	4	0	1.1	0.7
DO	9	5	3	3	3	2	3	1	0.6	0.8
FFC	0	14	0	6	0	5	0	4	NA	NA
SXT	11	3	6	0	5	0	0	4	16.1	0.001
E	13	1	6	0	5	0	4	0	1.1	0.7
CT	12	2	6	0	4	1	4	0	1.8	0.5
AMP	14	0	5	1	5	0	4	0	3.9	0.2
N	2	12	1	5	1	4	4	0	12.2	0.006
L	12	2	6	0	4	1	4	0	1.8	0.5
CE	13	1	6	0	5	0	4	0	1.1	0.7
SH	7	7	4	2	0	5	2	2	5.4	0.1
ENR	0	14	0	6	0	5	0	4	NA	NA
*mcr-*1	5	9	4	2	2	3	1	3	2.2	0.5
*sul-*1	6	8	3	3	2	3	0	4	2.9	0.3
*dfr*A1	5	9	2	4	3	2	1	3	1.4	0.7
*blaROB-1*	5	9	5	1	3	2	1	3	4.9	0.1
*erm*X	7	7	3	3	5	0	2	2	4.2	0.2
*tet*H	4	10	2	4	1	4	1	3	0.2	0.9
*pfh*A	6	8	5	1	2	3	2	2	3.1	0.3
*ptf*A	14	0	6	0	5	0	4	0	NA	NA
*fim*A	7	7	4	2	3	2	2	2	0.5	0.9
*exb*B	10	4	4	2	3	2	3	1	0.3	0.9
*pm*HAS	12	2	4	2	3	2	2	2	2.7	0.4
*omp*A	6	8	2	4	4	1	1	3	3.4	0.3
*tox*A	1	13	1	5	1	4	0	4	1.3	0.7
*ompH*	10	4	5	1	4	1	3	1	0.3	0.9
*soda*	14	0	6	0	5	0	4	0	NA	NA
*sod*C	12	2	6	0	5	0	4	0	2.3	0.5
*hgb*A	12	2	2	4	1	4	4	0	11.9	0.007
*nan*H	5	9	6	0	4	1	0	4	12.9	0.004
*oma*87	14	0	6	0	5	0	4	0	NA	NA

*AK* amikacin; *N* neomycin; *AMC* amoxicillin-clavulanic acid, *AMP* ampicillin, *CE* cephradine; *DO* doxycycline; *FFC* florfenicol; *SXT* sulfamethoxazole-trimethoprim; *CT* colistin, *E* erythromycin; *L* lincomycin; *SH* spectinomycin and *ENR* enrofloxacin

*p-values refer to significance of differences among studied animal species using Chi-square test. *p*-value cutoff was 0.05.

The *oma87, sodA, *and *ptfA* virulence genes were found in all (100%) examined avian species. However, *sodC* was detected in all isolates recovered from ducks. Furthermore, *sodC* and *hgbA* were found in turkeys and chickens with percentages of 100 and 80%, respectively. The *nanH* and *toxA* genes were not exhibited in any turkey isolates.

**Fig. 7:**
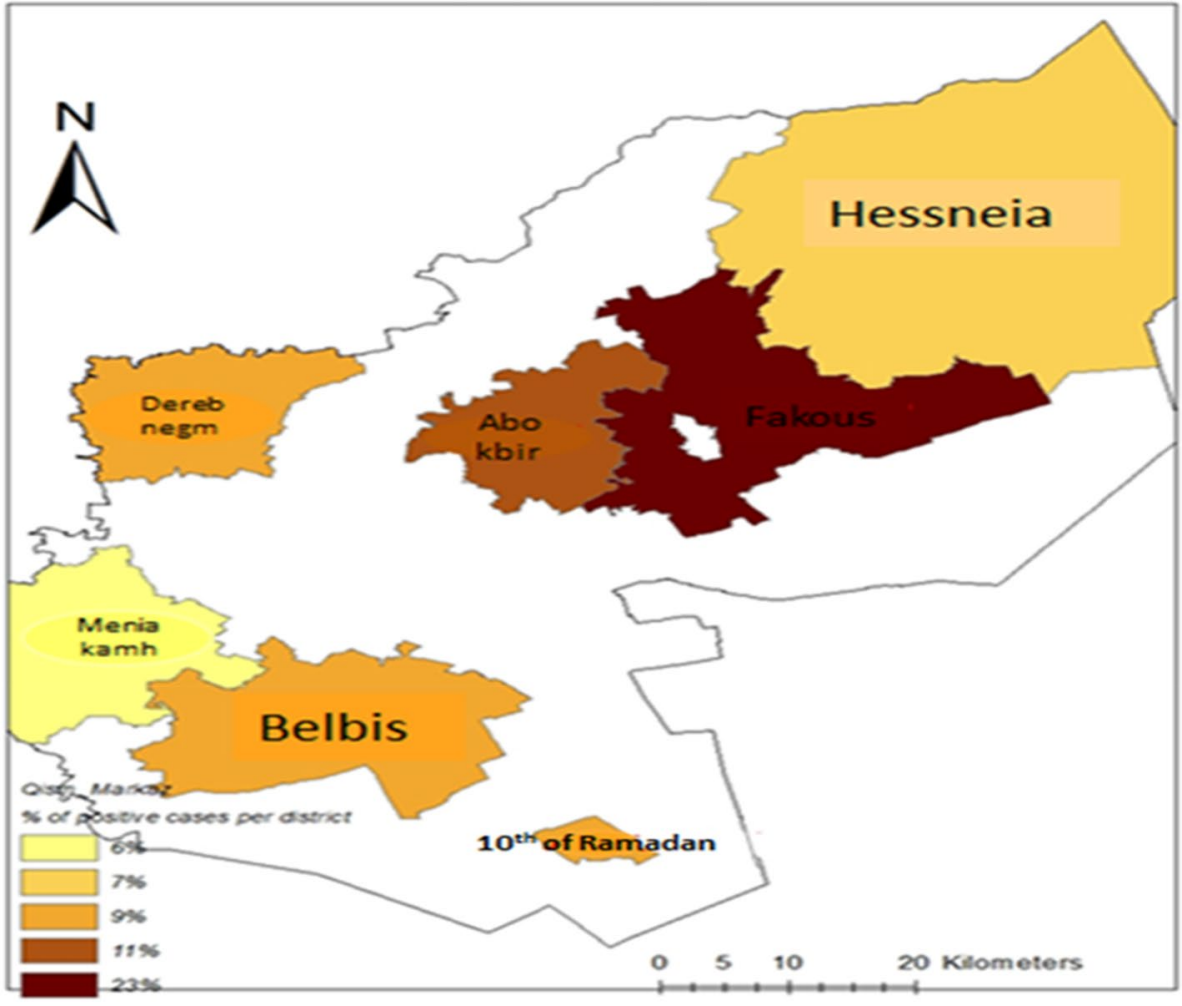
Epidemiological mapping of positive cases of *Pasteurellosis* in different districts in Sharkia Governorate, Egypt. Colors indicate number of positive cases per district*;* the more intense the color, the higher the occurrence

The map in Fig. 7 shows that the highest number of cases of pasteurellosis is in the Fakous district followed by the Abo kbir, Belbis, and Dereb negm districts.

## Discussion

Pasteurellosis has long been regarded as a serious economic burden on birds and other live animals. Due to its variety of clinical symptoms and time-consuming laboratory investigations, pasteurellosis diagnosis can be challenging (Abbas et al. 2018).

Studies of pathognomonic features of *P. multocida* isolates among various avian species have shown significant genetic variations among *P. multocida* strains from each avian species (Hurtado et al. 2020). These variations were attributed to many factors, including difference in host immune systems, various environmental conditions, and different transmission routes which have implications for the pathogenicity, diagnosis, treatment, and prevention of *P. multocida* infections in avian species (Saha et al. 2021; Shalaby et al. 2021).

Accompanying respiratory signs can reduce the feed intake leading to weight loss with the rapid progression of the disease. Congested catarrhal tracheitis with yellowish exudates is in harmony with those obtained by Marien (2007). Edematous firm yielded blood-cut surface-tinged exudate and mild hemorrhage was detected in *P*. *multocida* infection in complete accordance with those mentioned by Lakshman et al. (2006).

The tracheal lesions exhibited no characteristic lesion specified by the isolated strains in all examined birds. Detected lesions in ducks were more severe than those observed in chicken, quails, and turkeys which was similar to that reported by Awadin et al. (2017), however, we disagree with their findings regarding multiple granulomata in* P*. *multocida* infection.

Acute interstitial pneumonia was the most prominent lesion observed in acute respiratory infection of the lungs closely related to that described by Lakshman et al. (2006) who described the same lesion characterized by thickening of interalveolar septa with fibrin threads accumulation. Hemorrhages, congestion, and pneumonic changes were in line with the findings of Cynthia and Kahn (2005) and Ram and Abraham (2013) in the case of avian pasteurellosis.

In this study, *P. multocida* was isolated with a total prevalence of 9.4% (30/317) from the tested birds. The majority were of capsular type A (96.6%; 29/30). This is consistent with other studies (Mohamed and Mageed 2014; Abd-Elsadek et al. 2021; Shalaby et al. 2021) which isolated *P. multocida* in nearly similar percentages and found capsular type A to be the most common capsular type among avian strains. Interestingly, capsular type D was detected in 3.4% (1/30) of isolated *P. multocida* from quails, a serogroup considered rare (Glisson et al. 2008), demonstrating a variable prevalence of the serogroups in cases of respiratory disease according to the geographic region (Davies et al. 2003).

Climate change is a major environmental factor that can affect the transmission of this rare serogroup and wide spread of this infectious disease (Lafferty 2009; Bartlow et al. 2019)*. Pasteurella multocida* is more likely to survive and spread in warm, moist conditions (Iverson et al. 2016). As a result, climate change is expected to lead to an increase in the isolation rate of *P. multocida* isolates among various avian species.

A study published in 2017 found that the isolation rate of *P. multocida* isolates from poultry flocks in the United States increased by 20% between 1996 and 2016 (Nhung et al. 2017). The study also found that the increase in isolation rate was more pronounced in warmer regions of the country.

Another study, published in 2020 found that the survival of *P. multocida* in water droplets was significantly longer at temperatures of 37 °C and 42 °C than at 25 °C (Van Driessche et al. 2020). This suggests that climate change could lead to an increase in the transmission of *P. multocida* through contaminated water.

The impact of climate change on the isolation rate of *P. multocida* isolates is likely to vary depending on the specific region and the species of bird, however, the overall trend is expected to be an increase in isolation rate, as warmer temperatures and more extreme weather events create more favorable conditions for the bacterium to survive and spread (Wilson and Ho 2013).

In addition to climate change, other factors that can affect the isolation rate of *P. multocida* isolates include the density of bird populations, the presence of stress factors, such as overcrowding or poor nutrition, and the vaccination status of the birds (Iverson 2015).

The study also found that the breed, age, location, clinical symptoms, and sample type all had a substantial impact on the incidence rate of *P. multocida* in all hosts suggesting that several factors can contribute to the transmission and spread of *P. multocida* infections in avian species and give obvious explanations of the obtained prevalence rates.

Thirteen crucial virulence-associated genes (*pfhA, ptfA, fimA, exhB, pmHAS, ompA, ompH, toxA, hgbA, sodA, sodC, nanH,* and *oma87*) involved in pathogenesis (Aski and Tabatabaei 2016) were tested for presence in *P. multocida* isolates. According to the study's findings, all of the isolates had virulence-associated genes with various degrees.

It is well known that the *toxA* gene, which encodes the PMT-*P. multocida* toxin, is substantially connected to serogroup D. This toxin causes osteolysis, a crucial phase that follows the respiratory syndrome (Davies et al. 2003).

The primary requirement for bacterial infection is their attachment to the host cell, thus adhesion is regarded as one of the potential virulence factors (Sarangi et al. 2014 and Essawi et al. 2020). The high prevalence of several adhesion-related genes, including *fimA, pfhA,* and *ptfA*, was found in this study, indicating that either proteins operate synergistically or are necessary at various phases of colonization or infection. Filamentous hemagglutinins encoded by the *pfhA* gene play an important role in the initial colonization of the upper respiratory tract and the frequency of this gene varies greatly among strains of *P. multocida*. It has been established that this gene is a significant epidemiological marker and its prevalence among 50% of the obtained isolates is connected to the development of disease in several avian species (Haghnazari et al. 2017; Li et al. 2018). Interestingly, the absolute incidence of *ptfA* was recorded at 100% irrespective of its capsular type. These data were in the line with several prior studies (Sarangi et al. 2015; Furian et al. 2016; Vu-Khac et al. 2020).

Furthermore, the porin genes *ompA, ompH,* and *oma87* were found in 46.6%, 76.6%, and 100% of the isolates, respectively. These results imply that OMPs play a major role in host–pathogen interaction (He et al. 2021).

Hemoglobin binding protein-encoding gene (*hgbA*), neuraminidase gene (*nanH*), iron acquisition protein (*exhB*), and superoxide dismutase (*sodA, sodC*) were detected with 66.6, 50, 70, 100, and 93% prevalence, respectively. Differences in virulence profiles between investigations revealed that various *P. multocida* isolates may have diverse pathogenic mechanisms (Li et al. 2018; Abd El-Hamid et al. 2019; El-Demerdash et al. 2023b).

Diverse virulence profiles were detected for each isolate in correlation with their host or species, indicating differences in the *P. multocida* strains' capacity for invasion (Prajapati et al. 2020; El Damaty et al. 2023).

High frequencies of resistant genes were noticed towards erythromycin, β-lactam, and colistin in all species of examined birds. These results are not surprising as the isolates showed high resistance to erythromycin, β-lactam, and colistin antimicrobial drugs in susceptibility assays, which is comparable with prior findings in Egypt (Shalaby et al. 2021), Brazil (de Alcântara et al. 2020) and India (Sivagami et al. 2020), but our results were more sever and higher in rates. Additionally, the *dfrA1* gene which represents a resistance pattern to trimethoprim was detected at a higher rate in quail species.

 Presence of these genes on plasmids poses a great hazard to public health as bacteria can acquire a single plasmid to become multi-antibiotic resistant all at once, eventually developing multidrug resistance. Furthermore, plasmids frequently contain genes that affect the pathogenicity of bacteria (Cao et al. 2020), which explains the obtained high values of multiple resistance indices among various isolates and the appearance of extreme drug resistance (XDR) phenomenon.

The XDR of *P. multocida* in poultry is a serious threat to animal and public health as it is so difficult to treat infected birds and can lead to high mortality rates (Anholt et al. 2017; Algammal et al. 2022b).

Furthermore, it can be transmitted to humans, either through contact with infected animals or through the consumption of contaminated food posing a serious health risk, as it can cause severe infections, such as pneumonia and meningitis (Phillips et al. 2004).

There are a number of factors that can contribute to the emergence of XDR *P. multocida* in poultry including the overuse and misuse of antibiotics, the inadequate biosecurity measures, and the transportation and movement of birds between farms (Elayaraja et al. 2020; Bester et al. 2022).

The data have provided valuable insights into the association of antimicrobial resistance and virulence features in *P. multocida* isolates from various avian species. No particular clustering of analyzed genes or phenotypes was observed suggesting that the presence of antimicrobial resistance and virulence genes is not necessarily correlated. However, the study did find that there was a high positive significant correlation between lincomycin and colistin-resistant phenotypes. This suggests that the presence of one of these resistance genes may increase the likelihood of the presence of the other and a broad-spectrum approach to antimicrobial treatment may be necessary.

The study found that the frequency of occurrences of *sodA, ompH, ermX*, and *blaROB-1* genes is the most prevalent, especially in quails higher than in other understudied. This is attributed to many factors, such as the high density of these birds in commercial poultry farms, the use of antimicrobials in poultry farming, and poor hygiene practices used in the handling of birds.

In total, this study not only helped to represent a complete picture of the diagnosis of pasteurellosis but also aided in understanding the epidemiology of *P. multocida* infections among avian species.

Notably, the high number of cases in the Fakous district is likely due to several factors, including; the high density of livestock in the district, poor hygiene practices used in the handling of livestock, lack of access to clean water and sanitation and recent climate changes.

These findings highlight the need for interventions to improve hygiene practices used in livestock handling and to improve access to clean water and sanitation in the Fakous district, which could help to reduce the number of cases of pasteurellosis in the district.

Moreover, the obtained different pathogenic and genetic profiles of the isolates can help in the development of molecular diagnostic tests and vaccines for *P. multocida* infections in the poultry industry.

## Conclusion

The present study provides comprehensive epidemiological information on the diversity, histopathological, and virulence gene properties of Egyptian *Pasteurella multocida* isolates among various avian species. Moreover, the obtained data highlights the growing threat of factors that affect the isolation rate of *P. multocida* and antimicrobial resistance in examined bird species. The increasing prevalence of antimicrobial resistance and virulence genes in *P. multocida* observed during this study is a serious public health concern. Therefore, this research is essential to develop effective strategies to prevent the spread and control avian cholera, other diseases, and antimicrobial resistance caused by this bacterium, thus contributing to the great protection of human health.

### Supplementary Information

Below is the link to the electronic supplementary material.

Supplementary file1 (XLSX 32 KB)

## Data Availability

All data used have been included in the manuscript.

## References

[CR1] Abbas AM, Abd El-Moaty DAM, Zaki ESA (2018). Use of molecular biology tools for rapid identification and characterization of *Pasteurella* spp.. Vet World.

[CR2] Abd El-Hamid MI, El-Sayed ME, Ali AR (2019). Marjoram extract down-regulates the expression of *Pasteurella multocida* adhesion, colonization and toxin genes: a potential mechanism for its antimicrobial activity. Comp Immunol Microbiol Infect Dis.

[CR3] Abd-Elsadek E, Mostafa AEH, Abouelkhair A (2021). Molecular studies on *Pasteurella multocida* in ducks. J Curr Vet Res.

[CR4] Abdolmaleki Z, Mashak Z, Safarpoor Dehkordi F (2019). Phenotypic and genotypic characterization of antibiotic resistance in the methicillin-resistant *Staphylococcus aureus* strains isolated from hospital cockroaches. Antimicrob Resist Infect Control.

[CR5] Algammal AM, Hashem HR, Al-Otaibi AS (2021). Emerging MDR-*Mycobacterium avium* subsp. *avium* in house-reared domestic birds as the first report in Egypt. BMC Microbiol.

[CR6] Algammal AM, Abo Hashem ME, Alfifi KJ (2022). Sequence analysis, antibiogram profile, virulence and antibiotic resistance genes of XDR and MDR *Gallibacterium anatis* isolated from layer chickens in Egypt. Infect Drug Resist.

[CR7] Algammal AM, Ibrahim RA, Alfifi KJ (2022). A first report of molecular typing, virulence traits, and phenotypic and genotypic resistance patterns of newly emerging XDR and MDR *Aeromonas veronii* in *Mugil seheli*. Pathogens.

[CR8] Algammal AM, Eidaroos NH, Alfifi KJ (2023). oprL gene sequencing, resistance patterns, virulence genes, quorum sensing and antibiotic resistance genes of XDR *Pseudomonas aeruginosa* isolated from broiler chickens. Infect Drug Resist.

[CR9] Anholt RM, Klima C, Allan N (2017). Antimicrobial susceptibility of bacteria that cause bovine respiratory disease complex in Alberta. Canada Front Vet Sci.

[CR10] Apinda N, Nambooppha B, Rittipornlertrak A (2020). Protection against fowl cholera in ducks immunized with a combination vaccine containing live attenuated duck enteritis virus and recombinant outer membrane protein H of *Pasteurella multocida*. Avian Pathol.

[CR11] Aski HS, Tabatabaei M (2016). Occurrence of virulence-associated genes in *Pasteurella multocida* isolates obtained from different hosts. Microb Pathog.

[CR12] Awadin W, Ghaly S, Elsawak A (2017). Pathological and immunohistochemical study of *P. Multocida* capsular type a in tissues of chickens and ducks infected with fowl cholera. Assiut Vet Med J.

[CR13] Bartlow AW, Manore C, Xu C (2019). Forecasting zoonotic infectious disease response to climate change: mosquito vectors and a changing environment. Vet Sci.

[CR14] Bauer AW, Kirby WM, Sherris JC, Turck M (1966). Antibiotic susceptibility testing by a standardized single disk method. Am J Clin Pathol.

[CR15] Bester C, Marschik T, Schmoll F, Käsbohrer A (2022) Development of an economic model to assess the cost-effectiveness of biosecurity measures to reduce the burden of Salmonella and Hepatitis E virus in the pork production chain. In: 20th Congress of the International Society for Animal Hygiene. p 107

[CR16] Cao Y-P, Lin Q-Q, He W-Y (2020). Co-selection may explain the unexpectedly high prevalence of plasmid-mediated colistin resistance gene *mcr*-1 in a Chinese broiler farm. Zool Res.

[CR17] Carter GR (1984) Pasteurella. Bergeys Manual of Systematic Bacteriology. Williams and Wilkins (Krieg, N R and J G Holt, Eds), Baltimore, 1: 552–557

[CR18] Christenson ES, Ahmed HM, Durand CM (2015). *Pasteurella multocida* infection in solid organ transplantation. Lancet Infect Dis.

[CR19] CLSI (2020) CLSI M100-ED29: 2021 Performance Standards for Antimicrobial Susceptibility Testing, 30th Edition. Clsi 40:50–51

[CR20] Corchia A, Limelette A, Hubault B (2015). Rapidly evolving conjunctivitis due to *Pasteurella multocida*, occurring after direct inoculation with animal droplets in an immuno-compromised host. BMC Ophthalmol.

[CR21] Cynthia MK, Kahn M (2005). The Merck veterinary manual.

[CR22] Davies RL, MacCorquodale R, Caffrey B (2003). Diversity of avian *Pasteurella multocida* strains based on capsular PCR typing and variation of the *Omp*A and *Omp*H outer membrane proteins. Vet Microbiol.

[CR23] de Alcântara RI, Ferrari RG, Panzenhagen PHN (2020). Antimicrobial resistance genes in bacteria from animal-based foods. Adv Appl Microbiol.

[CR24] de Cecco BS, Carossino M, Del Piero F (2021). Meningoencephalomyelitis in domestic cats: 3 cases of *Pasteurella multocida* infection and literature review. J Vet Diagn Invest.

[CR25] Ebrahem AF, El-Demerdash AS, Orady RM, Nabil NM (2023). Modulatory effect of competitive exclusion on the transmission of ESBL *E. coli* in chickens. Probiotics Antimicrob Proteins.

[CR26] El Damaty HM, El-Demerdash AS, Abd El-Aziz NK (2023). Molecular characterization and antimicrobial susceptibilities of *Corynebacterium pseudotuberculosis* Isolated from caseous lymphadenitis of smallholder sheep and goats. Animals.

[CR27] Elayaraja S, Mabrok M, Algammal A (2020). Potential influence of jaggery-based biofloc technology at different C: N ratios on water quality, growth performance, innate immunity, immune-related genes expression profiles, and disease resistance against *Aeromonas hydrophila* in *Nile tilapia* (Oreochro. Fish Shellfish Immunol.

[CR28] El-Demerdash AS, Aggour MG, El-Azzouny MM, Abou-Khadra SH (2018). Molecular analysis of integron gene cassette arrays associated multi-drug resistant Enterobacteriaceae isolates from poultry. Cell Mol Biol.

[CR29] El-Demerdash AS, Bakry NR, Aggour MG (2023). *Bovine* Mastitis in Egypt: bacterial etiology and evaluation of diagnostic biomarkers. Int J Vet Sci.

[CR30] El-Demerdash AS, Mohamady SN, Megahed HM, Ali NM (2023). Evaluation of gene expression related to immunity, apoptosis, and gut integrity that underlies Artemisia’s therapeutic effects in necrotic enteritis-challenged broilers. 3 Biotech.

[CR31] Essawi WM, El-Demerdash AS, El-Mesalamy MM, Abonorag MA (2020). Validation of camel’s fetal fluids as antimicrobial agents. Curr Microbiol.

[CR32] Furian TQ, Borges KA, Laviniki V (2016). Virulence genes and antimicrobial resistance of *Pasteurella multocida* isolated from poultry and swine. Braz J Microbiol.

[CR33] Glisson JR, Sandhu TS, Hofacre CL (2008) Pasteurellosis, avibacteriosis, gallibacteriosis, riemerellosis and pseudotuberculosis. A laboratory manual for the isolation, identification and characterization of avian pathogens Georgia: American Association of Avian Pathologists 12–18

[CR34] Haghnazari S, Jabbari AR, Tadayon K (2017). Prevalence of adhesion virulence factor genes, antibiogram, and pathogenicity of avian *Pasteurella multocida* isolate from Iran. Arch Razi Inst.

[CR35] Harper M, Boyce JD, Adler B (2006). *Pasteurella multocida* pathogenesis: 125 years after Pasteur. FEMS Microbiol Lett.

[CR36] Harper M, St Michael F, Steen JA (2015). Characterization of the lipopolysaccharide produced by *Pasteurella multocida* serovars 6, 7 and 16: identification of lipopolysaccharide genotypes L4 and L8. Glycobiology.

[CR37] He F, Zhao Z, Wu X (2021). Transcriptomic analysis of high-and low-virulence *Bovine*
*Pasteurella multocida* in vitro and in vivo. Front Vet Sci.

[CR38] Heddleston KL, Rebers PA (1975). Properties of free endotoxin from *Pasteurella multocida*. Am J Vet Res.

[CR39] Heuer H, Smalla K (2007). Manure and sulfadiazine synergistically increased bacterial antibiotic resistance in soil over at least 2 months. Environ Microbiol.

[CR40] Hurtado R, Maturrano L, Azevedo V, Aburjaile F (2020). Pathogenomics insights for understanding *Pasteurella multocida* adaptation. Int J Med Microbiol.

[CR41] Iverson S (2015). Quantifying the demographic and population impact of avian cholera on Northern Common Eiders in the face of ancillary threats and changing environmental circumstances.

[CR42] Iverson SA, Forbes MR, Simard M (2016). Avian cholera emergence in arctic-nesting northern Common Eiders: using community-based, participatory surveillance to delineate disease outbreak patterns and predict transmission risk. Ecol Soc.

[CR43] Klima CL, Alexander TW, Hendrick S, McAllister TA (2014). Characterization of *Mannheimia haemolytica* isolated from feedlot cattle that were healthy or treated for bovine respiratory disease. Can J Vet Res.

[CR44] Kolde R (2012) Pheatmap: pretty heatmaps. R package version 1:

[CR45] Lafferty KD (2009). The ecology of climate change and infectious diseases. Ecology.

[CR46] Lakshman M, Shashikumar M, Rajendranath N (2006) Pathology of lung affections in poultry-A field study Indian J Vet Pathol. 30(1):42–45

[CR47] Li Z, Cheng F, Lan S (2018). Investigation of genetic diversity and epidemiological characteristics of *Pasteurella multocida* isolates from poultry in southwest China by population structure, multi-locus sequence typing and virulence-associated gene profile analysis. J Vet Med Sci.

[CR48] Liu H, Zhao Z, Xi X (2017). Occurrence of *Pasteurella multocida* among pigs with respiratory disease in China between 2011 and 2015. Ir Vet J.

[CR49] Lopez A, Martinson SA (2017). Respiratory system, mediastinum, and pleurae. Pathol Basis Vet Dis.

[CR50] Magiorakos A-P, Srinivasan A, Carey RB (2012). Multidrug-resistant, extensively drug-resistant and pandrug-resistant bacteria: an international expert proposal for interim standard definitions for acquired resistance. Clin Microbiol Infect.

[CR51] Marien M (2007). Mixed respiratory infections in turkeys, with emphasis on avian *metapneumovirus, Ornithobacterium rhinotracheale, Escherichia coli and Mycoplasma gallisepticum*.

[CR52] Markey, B.; Leonard, F.; Archambault, M.; Cullinane, A. and Maguire D (2013) Clinical veterinary microbiology. second Ed MOSBY ELSEVIER Chapter 21:307- 316

[CR53] Martins IR (2022) Tell Me How You Look Like And I’ll Tell You Where You Come From: Identification Of Bivalves Shells’ Origin. PhD Thesis

[CR54] Megahed MMM, El-Nagar AMA, El-Demerdash AS (2023). Evaluation and development of diagnostic tools for rapid detection of *Riemerella anatipestifer* and *Pasteurella multocida* in ducks. J Adv Vet Anim Res.

[CR55] Mohamed M-WA, Mageed MAAMA (2014). Molecular analysis of *Pasteurella multocida* strains isolated from fowl cholera infection in backyard chickens. Asian Pac J Trop Biomed.

[CR100] Nhung NT, Chansiripornchai N, Carrique-Mas JJ (2017) Antimicrobial resistance in bacterial poultry pathogens: a review. Front Vet Sci 4: 126. 10.3389/fvets.2017.00126 PMC5554362 28848739

[CR56] Panna S, Nazir K, Rahman M (2015). Isolation and molecular detection of *Pasteurella multocida* Type A from naturally infected chickens, and their histopathological evaluation in artificially infected chickens in Bangladesh. J Adv Vet Anim Res.

[CR57] Peng Z, Liang W, Wu B (2016). Molecular typing methods for *Pasteurella multocida*-a review. Wei Sheng Wu Xue Bao.

[CR58] Phillips I, Casewell M, Cox T (2004). Does the use of antibiotics in food animals pose a risk to human health? a critical review of published data. J Antimicrob Chemother.

[CR59] Prajapati A, Chanda MM, Yogisharadhya R (2020). Comparative genetic diversity analysis based on virulence and repetitive genes profiling of circulating *Pasteurella multocida* isolates from animal hosts. Infect Genet Evol.

[CR60] Quinn PJ, Markey BK, Leonard FC (2011). Veterinary microbiology and microbial disease.

[CR61] Ram A, Abraham MJ (2013) Histopathological lesions in pasteurellosis in an emu (Dromaius novaehollandiae)-a case report

[CR62] Rosato AE, Lee BS, Nash KA (2001). Inducible macrolide resistance in *Corynebacterium jeikeium*. Antimicrob Agents Chemother.

[CR63] Saha O, Islam MR, Rahman MS (2021). First report from Bangladesh on genetic diversity of multidrug-resistant *Pasteurella multocida* type B: 2 in fowl cholera. Vet World.

[CR64] Sarangi LN, Priyadarshini A, Kumar S (2014). Virulence genotyping of *Pasteurella multocida* isolated from multiple hosts from India. Sci World J.

[CR65] Sarangi LN, Thomas P, Gupta SK (2015). Virulence gene profiling and antibiotic resistance pattern of Indian isolates of *Pasteurella multocida* of small ruminant origin. Comp Immunol Microbiol Infect Dis.

[CR66] Shalaby AG, Bakry NR, El-Demerdash AS (2021). Virulence attitude estimation of *Pasteurella multocida* isolates in embryonated chicken eggs. Arch Microbiol.

[CR67] Sid H, Benachour K, Rautenschlein S (2015). Co-infection with multiple respiratory pathogens contributes to increased mortality rates in Algerian poultry flocks. Avian Dis.

[CR68] Sivagami K, Vignesh VJ, Srinivasan R (2020). Antibiotic usage, residues and resistance genes from food animals to human and environment: an Indian scenario. J Environ Chem Eng.

[CR69] Suvarna SK, Layton C, Bancroft JD (2013) Theory and practice of histological techniques. Pbl London, Churchill Livingstone, Elsiver 173–187

[CR70] Tambekar DH, Dhanorkar D V, Gulhane SR, et al (2006) Antibacterial susceptibility of some urinary tract pathogens to commonly used antibiotics. Afr J Biotechnol 5:

[CR71] Tang X, Zhao Z, Hu J (2009). Isolation, antimicrobial resistance, and virulence genes of *Pasteurella multocida* strains from swine in China. J Clin Microbiol.

[CR72] Townsend KM, Frost AJ, Lee CW (1998). Development of PCR assays for species-and type-specific identification of *Pasteurella multocida* isolates. J Clin Microbiol.

[CR73] Townsend KM, Boyce JD, Chung JY (2001). Genetic organization of *Pasteurella multocida* cap loci and development of a multiplex capsular PCR typing system. J Clin Microbiol.

[CR74] Van Driessche L, De Neve C, Haesebrouck F (2020). Storage time and temperature affect the isolation rate of *Mannheimia haemolytica* and *Pasteurella multocida* from bovine bronchoalveolar lavage samples. BMC Vet Res.

[CR75] Vickers NJ (2017). Animal communication: when I’m calling you, will you answer too?. Curr Biol.

[CR76] Vu-Khac H, Trinh TTH, Nguyen TTG (2020). Prevalence of virulence factor, antibiotic resistance, and serotype genes of *Pasteurella multocida* strains isolated from pigs in Vietnam. Vet World.

[CR77] Wang C, Wu Y, Xing X (2009). An outbreak of avian cholera in wild waterfowl in Ordos wetland, Inner Mongolia, China. J Wildl Dis.

[CR78] Wickham H (2009). Elegant graphics for data analysis. Media.

[CR79] Wilson BA, Ho M (2013). *Pasteurella multocida*: from zoonosis to cellular microbiology. Clin Microbiol Rev.

[CR80] Wilson MA, Morgan MJ, Barger GE (1993). Comparison of DNA fingerprinting and serotyping for identification of avian *Pasteurella multocida* isolates. J Clin Microbiol.

[CR81] Wilson M, Henderson B, McNab R (2002). Bacterial disease mechanisms: an introduction to cellular microbiology.

[CR82] Zou D, Huang S, Lei H (2017). Sensitive and rapid detection of the plasmid-encoded colistin-resistance gene *mcr-1* in Enterobacteriaceae isolates by loop-mediated isothermal amplification. Front Microbiol.

